# Ethyl 2-hydr­oxy-5-oxo-4-phenyl-2,3,4,5-tetra­hydro­pyrano[3,2-*c*]chromene-2-carboxyl­ate

**DOI:** 10.1107/S1600536809028311

**Published:** 2009-07-25

**Authors:** Wei Zhang, Guangcun Zhang, Bailin Li, Yifeng Wang

**Affiliations:** a State Key Laboratory Breeding Base of Green Chemistry-Synthesis Technology, Zhejiang University of Technology, Hangzhou 310014, People’s Republic of China; b Department of Pharmaceutical and Chemical Engineering, Taizhou College, Linhai, Zhejiang 317000, People’s Republic of China

## Abstract

The main structural unit of the title compoud, C_21_H_18_O_6_, is a fused three-ring group consisting of coumarin and tetra­hydro­pyrane ring systems. Two C atoms of the tetra­hydro­pyran ring are displaced by 0.295 (3) and −0.360 (2) Å from the mean plane of coumarin ring. The dihedral angle between the phenyl and coumarin rings is 73.94 (3)°. Inter­molecular O—H⋯O hydrogen bonds are present in the crystal structure.

## Related literature

For the synthesis of (*E*)-ethyl 2-oxo-4-phenyl­but-3-enoate, see: Vaijayanthi & Chadha (2007 ▶).
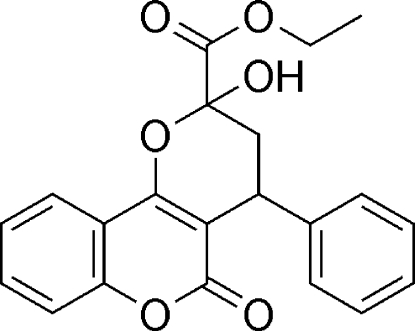


## Experimental

### 

#### Crystal data

C_21_H_18_O_6_
*M* *_r_* = 366.37
Monoclinic, 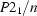

*a* = 5.4988 (2) Å
*b* = 14.9975 (5) Å
*c* = 21.342 (1) Å
β = 98.5487 (13)°
*V* = 1740.48 (12) Å^3^
*Z* = 4
Mo *K*α radiation
μ = 0.10 mm^−1^
*T* = 296 K
0.41 × 0.39 × 0.14 mm

#### Data collection

Rigaku R-AXIS RAPID diffractometer
Absorption correction: multi-scan (*ABSCOR*; Higashi, 2005 ▶) *T*_min_ = 0.954, *T*_max_ = 0.986
16523 measured reflections
3422 independent reflections
2568 reflections with *F* ^2^ > 2σ(*F* ^2^)
*R*_int_ = 0.026

#### Refinement

*R*[*F* ^2^ > 2σ(*F* ^2^)] = 0.035
*wR*(*F* ^2^) = 0.080
*S* = 1.00
3422 reflections
246 parameters
H-atom parameters constrained
Δρ_max_ = 0.21 e Å^−3^
Δρ_min_ = −0.19 e Å^−3^

### 

Data collection: *PROCESS-AUTO* (Rigaku, 2006 ▶); cell refinement: *PROCESS-AUTO*; data reduction: *CrystalStructure* (Rigaku, 2007 ▶); program(s) used to solve structure: *SHELXL97* (Sheldrick, 2008 ▶); program(s) used to refine structure: *SHELXL97* (Sheldrick, 2008 ▶); molecular graphics: *ORTEP-3 for Windows* (Farrugia, 1997 ▶); software used to prepare material for publication: *CrystalStructure*.

## Supplementary Material

Crystal structure: contains datablocks global, I. DOI: 10.1107/S1600536809028311/pk2181sup1.cif

Structure factors: contains datablocks I. DOI: 10.1107/S1600536809028311/pk2181Isup2.hkl

Additional supplementary materials:  crystallographic information; 3D view; checkCIF report

## Figures and Tables

**Table 1:** Hydrogen-bond geometry (Å, °)

*D*—H⋯*A*	*D*—H	H⋯*A*	*D*⋯*A*	*D*—H⋯*A*
O1—H101⋯O2^i^	0.82	2.30	2.9198 (15)	132
O1—H101⋯O5	0.82	2.17	2.6628 (17)	119

Symmetry code: (i) 
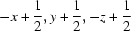
.

**Fig. 1.**
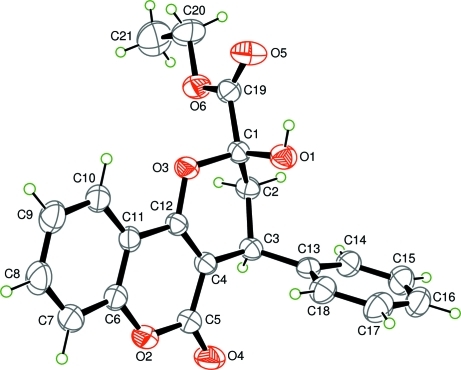
The asymmetric unit of the structure of the title compound, with the atomic labeling scheme. Displacement ellipsoids are drawn at the 50% probability level.

**Fig. 2.**
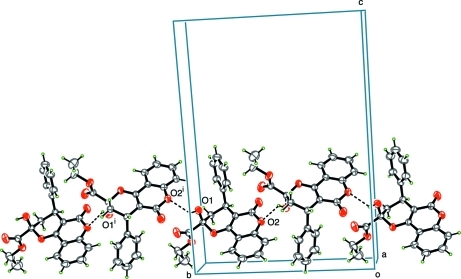
The molecular packing of the title compound showing H-bond interactions. Symmetry code (i) is 0.5-x, 0.5+y, 0.5-z.

## supplementary crystallographic information

### Comment

Coumarin derivatives are widely distributed in nature and are used as versatile intermediates in organic and natural product synthesis. Moreover, this class of compound possess a wide range of biological activities, including anticoagulant and HIV protease inhibition properties.  The title compound, which is readily synthesized from commercially available 4-hydroxycoumarin and (*E*)-ethyl 2-oxo- 4-phenylbut-3-enoate, can act as an intermediate in organic and natural product synthesis.  In this article, the crystal structure of the title compound, ethyl 2-hydroxy-5-oxo-4-phenyl-2,3,4,5-tetrahydropyrano[3,2- *c*]chromene-2-carboxylate was described (Fig. 1).  The main structural unit is a three-ring group consisting of a coumarin ring and a tetrahydropyrane.  Two carbon atoms of the tetrahydropyrane structure are not coplanar with the coumarin backbone: one carbon atom lies 0.295 (3) Å from the mean plane of coumarin ring and the other lies 0.360 (2) Å from the plane in opposite direction.  The dihedral angle between benzene and coumarin rings is 73.94 (3) °. The distance from O1 of the hydroxyl group to coumarin plane is 1.664 (2) Å.  In addition, intermolecular O—H···O hydrogen bonds in the crystal are observed (Fig. 2).

### Experimental

The title compound was synthesized by treating (*E*)-ethyl 2-oxo-4-phenylbut-3-enoate (2.04 g,10 mmol) with 4-hydroxycoumarin (1.62 g, 10 mmol) in the presence of triethylamine as a catalyst in dichloromethane (30 ml) under stirring at room temperature for 24 h. The solvent was distilled under vacuum, and the residue was purified by flash column chromatography (silica gel, Hex/AcOEt, *v*/*v*, 3:1) giving the title compound (3.3 g, 90%). The compound (*E*)-ethyl 2-oxo-4-phenylbut-3-enoate was obtained from commercially available benzaldehyde by condensation with pyruvic acid and subsequent esterification with ethanol. Suitable crystals of the title compound were obtained by slow evaporation of dichloromethane solution at room temperature.

### Refinement

H atoms were placed in calculated position with C—H=0.98 Å(*sp*), C—H=0.97 Å(sp2), C—H=0.96 Å(sp3), C—H=0.93 Å(aromatic). All H atoms included in the final cycles of refinement using a riding model, with *U*_iso_(H)=1.2*U*_eq_ of the carrier atoms.

### Crystal data

**Table:**

C_21_H_18_O_6_	*F*(000) = 768.00
*M**_r_* = 366.37	*D*_x_ = 1.398 Mg m^−^^3^
Monoclinic, *P*2_1_/*n*	Mo *K*α radiation, λ = 0.71075 Å
Hall symbol: -P 2yn	Cell parameters from 11365 reflections
*a* = 5.4988 (2) Å	θ = 3.2–27.4°
*b* = 14.9975 (5) Å	µ = 0.10 mm^−^^1^
*c* = 21.342 (1) Å	*T* = 296 K
β = 98.5487 (13)°	Platelet, colorless
*V* = 1740.48 (12) Å^3^	0.41 × 0.39 × 0.14 mm
*Z* = 4	

### Data collection

**Table:**

Rigaku R-AXIS RAPID diffractometer	2568 reflections with *F*^2^ > 2σ(*F*^2^)
Detector resolution: 10.00 pixels mm^-1^	*R*_int_ = 0.026
ω scans	θ_max_ = 27.4°
Absorption correction: multi-scan (*ABSCOR*; Higashi, 2005)	*h* = −7→7
*T*_min_ = 0.954, *T*_max_ = 0.986	*k* = −19→16
16523 measured reflections	*l* = −27→27
3422 independent reflections	

### Refinement

**Table:**

Refinement on *F*^2^	H-atom parameters constrained
*R*[*F*^2^ > 2σ(*F*^2^)] = 0.035	*w* = 1/[σ^2^(*F*_o_^2^) + (0.013*P*)^2^ + *P*] where *P* = (*F*_o_^2^ + 2*F*_c_^2^)/3
*wR*(*F*^2^) = 0.080	(Δ/σ)_max_ = 0.001
*S* = 1.00	Δρ_max_ = 0.21 e Å^−^^3^
3422 reflections	Δρ_min_ = −0.19 e Å^−^^3^
246 parameters	Extinction correction: *SHELXL97* (Sheldrick, 2008)
0 restraints	Extinction coefficient: 0.0057 (4)

### Special details

**Table:**

Geometry. The tetrahydropyrane structure in the crystal displays an envelope configuration, with atom C2 at the flap position, displaced by 0.603 (2) Å from the mean plane of the other atoms.
Refinement. Refinement using reflections with *F*^2^ > 2.0 σ(*F*^2^). The weighted *R*-factor(*wR*), goodness of fit (*S*) and *R*-factor (gt) are based on *F*, with *F* set to zero for negative *F*. The threshold expression of *F*^2^ > 2.0 σ(*F*^2^) is used only for calculating *R*-factor (gt).

### Fractional atomic coordinates and isotropic or equivalent isotropic displacement parameters (Å^2^)

**Table:**

	*x*	*y*	*z*	*U*_iso_*/*U*_eq_	
O1	0.3270 (2)	0.47260 (6)	0.24846 (5)	0.0400 (2)	
O2	0.3755 (2)	0.12605 (6)	0.19275 (5)	0.0442 (3)	
O3	0.2981 (2)	0.39483 (6)	0.15453 (5)	0.0369 (2)	
O4	0.7169 (2)	0.14798 (8)	0.25944 (6)	0.0548 (3)	
O5	0.2708 (2)	0.60253 (9)	0.16249 (6)	0.0562 (3)	
O6	0.5597 (2)	0.54329 (8)	0.11100 (6)	0.0483 (3)	
C1	0.4400 (2)	0.46019 (10)	0.19571 (6)	0.0320 (3)	
C2	0.7020 (2)	0.42864 (10)	0.21432 (8)	0.0344 (3)	
C3	0.7153 (2)	0.33839 (10)	0.24884 (6)	0.0326 (3)	
C4	0.5223 (2)	0.27767 (10)	0.21402 (6)	0.0322 (3)	
C5	0.5514 (3)	0.18281 (11)	0.22491 (8)	0.0383 (3)	
C6	0.1792 (3)	0.15776 (11)	0.15092 (6)	0.0390 (3)	
C7	0.0084 (3)	0.09575 (12)	0.12389 (8)	0.0504 (4)	
C8	−0.1910 (3)	0.12559 (12)	0.08244 (9)	0.0548 (5)	
C9	−0.2214 (3)	0.21535 (12)	0.06798 (8)	0.0490 (4)	
C10	−0.0505 (2)	0.27653 (11)	0.09506 (6)	0.0394 (3)	
C11	0.1532 (2)	0.24823 (10)	0.13760 (6)	0.0335 (3)	
C12	0.3355 (2)	0.30795 (10)	0.17075 (6)	0.0312 (3)	
C13	0.7091 (2)	0.34673 (10)	0.31968 (6)	0.0334 (3)	
C14	0.9040 (3)	0.38961 (12)	0.35684 (8)	0.0429 (4)	
C15	0.9097 (3)	0.39905 (12)	0.42137 (9)	0.0526 (4)	
C16	0.7198 (3)	0.36628 (13)	0.45022 (9)	0.0554 (5)	
C17	0.5260 (3)	0.32322 (13)	0.41407 (9)	0.0526 (4)	
C18	0.5209 (3)	0.31326 (12)	0.34917 (8)	0.0427 (4)	
C19	0.4127 (2)	0.54477 (11)	0.15467 (8)	0.0368 (3)	
C20	0.5607 (4)	0.62325 (13)	0.07224 (10)	0.0632 (5)	
C21	0.7368 (4)	0.60702 (17)	0.02723 (11)	0.0822 (7)	
H3	0.8750	0.3122	0.2443	0.039*	
H7	0.0279	0.0354	0.1335	0.060*	
H8	−0.3073	0.0848	0.0638	0.066*	
H9	−0.3574	0.2343	0.0400	0.059*	
H10	−0.0706	0.3367	0.0851	0.047*	
H11	0.7910	0.4727	0.2421	0.041*	
H12	0.7781	0.4226	0.1763	0.041*	
H14	1.0325	0.4123	0.3378	0.051*	
H15	1.0420	0.4276	0.4455	0.063*	
H16	0.7223	0.3731	0.4936	0.066*	
H17	0.3979	0.3007	0.4333	0.063*	
H18	0.3898	0.2838	0.3253	0.051*	
H101	0.2350	0.5159	0.2417	0.048*	
H201	0.6127	0.6743	0.0988	0.076*	
H202	0.3976	0.6341	0.0492	0.076*	
H211	0.8950	0.5924	0.0504	0.099*	
H212	0.7504	0.6597	0.0025	0.099*	
H213	0.6791	0.5585	−0.0003	0.099*	

### Atomic displacement parameters (Å^2^)

**Table:**

	*U*^11^	*U*^22^	*U*^33^	*U*^12^	*U*^13^	*U*^23^
O1	0.0426 (6)	0.0374 (6)	0.0417 (6)	0.0076 (5)	0.0117 (5)	0.0020 (5)
O2	0.0565 (7)	0.0292 (5)	0.0443 (6)	−0.0051 (5)	−0.0012 (5)	0.0017 (5)
O3	0.0413 (6)	0.0266 (5)	0.0394 (6)	−0.0019 (4)	−0.0047 (4)	0.0001 (4)
O4	0.0603 (8)	0.0355 (6)	0.0628 (8)	0.0082 (6)	−0.0107 (6)	0.0050 (6)
O5	0.0556 (7)	0.0415 (7)	0.0723 (9)	0.0130 (6)	0.0120 (6)	0.0145 (6)
O6	0.0652 (8)	0.0371 (6)	0.0445 (7)	−0.0020 (5)	0.0149 (6)	0.0096 (5)
C1	0.0357 (8)	0.0282 (7)	0.0322 (8)	−0.0027 (6)	0.0054 (6)	−0.0020 (6)
C2	0.0326 (7)	0.0315 (8)	0.0393 (8)	−0.0032 (6)	0.0056 (6)	0.0008 (6)
C3	0.0293 (7)	0.0318 (8)	0.0364 (8)	0.0025 (6)	0.0041 (6)	−0.0009 (6)
C4	0.0358 (8)	0.0281 (7)	0.0329 (8)	−0.0006 (6)	0.0059 (6)	−0.0011 (6)
C5	0.0457 (9)	0.0324 (8)	0.0365 (8)	−0.0014 (7)	0.0053 (7)	−0.0002 (7)
C6	0.0492 (9)	0.0373 (9)	0.0303 (8)	−0.0064 (7)	0.0049 (7)	−0.0005 (6)
C7	0.0719 (12)	0.0364 (9)	0.0404 (10)	−0.0173 (9)	0.0005 (9)	0.0006 (7)
C8	0.0683 (12)	0.0529 (11)	0.0398 (10)	−0.0267 (10)	−0.0031 (9)	−0.0026 (8)
C9	0.0507 (10)	0.0559 (11)	0.0376 (9)	−0.0111 (9)	−0.0025 (7)	−0.0013 (8)
C10	0.0457 (9)	0.0386 (9)	0.0333 (8)	−0.0048 (7)	0.0040 (7)	−0.0014 (7)
C11	0.0401 (8)	0.0331 (8)	0.0276 (7)	−0.0054 (7)	0.0064 (6)	−0.0032 (6)
C12	0.0370 (8)	0.0273 (7)	0.0304 (7)	−0.0023 (6)	0.0089 (6)	−0.0010 (6)
C13	0.0335 (7)	0.0303 (8)	0.0355 (8)	0.0065 (6)	0.0016 (6)	0.0027 (6)
C14	0.0405 (9)	0.0452 (10)	0.0408 (9)	0.0015 (7)	−0.0014 (7)	−0.0003 (7)
C15	0.0515 (10)	0.0561 (11)	0.0445 (10)	0.0083 (9)	−0.0117 (8)	−0.0060 (8)
C16	0.0647 (12)	0.0645 (12)	0.0351 (9)	0.0220 (10)	0.0014 (9)	−0.0008 (9)
C17	0.0525 (10)	0.0642 (12)	0.0433 (10)	0.0116 (9)	0.0146 (8)	0.0081 (9)
C18	0.0394 (9)	0.0465 (10)	0.0416 (9)	0.0015 (7)	0.0042 (7)	0.0010 (7)
C19	0.0398 (8)	0.0317 (8)	0.0372 (9)	−0.0057 (7)	0.0005 (7)	0.0013 (6)
C20	0.0818 (14)	0.0490 (11)	0.0601 (13)	−0.0083 (10)	0.0147 (11)	0.0222 (9)
C21	0.1051 (19)	0.0793 (16)	0.0674 (15)	−0.0195 (14)	0.0301 (14)	0.0151 (13)

### Geometric parameters (Å, °)

**Table:**

O1—C1	1.3772 (19)	C13—C18	1.383 (2)
O2—C5	1.3906 (19)	C14—C15	1.380 (2)
O2—C6	1.3791 (18)	C15—C16	1.379 (2)
O3—C1	1.4623 (17)	C16—C17	1.379 (2)
O3—C12	1.3560 (18)	C17—C18	1.389 (2)
O4—C5	1.2019 (19)	C20—C21	1.482 (3)
O5—C19	1.194 (2)	O1—H101	0.822
O6—C19	1.321 (2)	C2—H11	0.970
O6—C20	1.457 (2)	C2—H12	0.970
C1—C2	1.512 (2)	C3—H3	0.980
C1—C19	1.536 (2)	C7—H7	0.930
C2—C3	1.538 (2)	C8—H8	0.930
C3—C4	1.508 (2)	C9—H9	0.930
C3—C13	1.522 (2)	C10—H10	0.930
C4—C5	1.447 (2)	C14—H14	0.930
C4—C12	1.353 (2)	C15—H15	0.930
C6—C7	1.385 (2)	C16—H16	0.930
C6—C11	1.389 (2)	C17—H17	0.930
C7—C8	1.377 (2)	C18—H18	0.930
C8—C9	1.386 (2)	C20—H201	0.970
C9—C10	1.378 (2)	C20—H202	0.970
C10—C11	1.399 (2)	C21—H211	0.960
C11—C12	1.448 (2)	C21—H212	0.960
C13—C14	1.392 (2)	C21—H213	0.960
			
C5—O2—C6	121.90 (12)	O5—C19—C1	122.00 (16)
C1—O3—C12	116.34 (10)	O6—C19—C1	111.72 (13)
C19—O6—C20	116.16 (14)	O6—C20—C21	106.91 (17)
O1—C1—O3	108.45 (12)	C1—O1—H101	107.7
O1—C1—C2	110.84 (12)	C1—C2—H11	108.8
O1—C1—C19	109.68 (12)	C1—C2—H12	108.8
O3—C1—C2	110.52 (12)	C3—C2—H11	108.8
O3—C1—C19	102.28 (11)	C3—C2—H12	108.8
C2—C1—C19	114.63 (13)	H11—C2—H12	109.5
C1—C2—C3	112.15 (12)	C2—C3—H3	106.5
C2—C3—C4	108.40 (11)	C4—C3—H3	106.5
C2—C3—C13	113.39 (12)	C13—C3—H3	106.5
C4—C3—C13	114.91 (12)	C6—C7—H7	120.8
C3—C4—C5	117.50 (12)	C8—C7—H7	120.8
C3—C4—C12	122.74 (13)	C7—C8—H8	119.4
C5—C4—C12	119.62 (13)	C9—C8—H8	119.4
O2—C5—O4	116.37 (14)	C8—C9—H9	119.9
O2—C5—C4	118.06 (13)	C10—C9—H9	119.9
O4—C5—C4	125.57 (14)	C9—C10—H10	120.0
O2—C6—C7	117.07 (14)	C11—C10—H10	120.0
O2—C6—C11	121.07 (13)	C13—C14—H14	119.4
C7—C6—C11	121.85 (14)	C15—C14—H14	119.4
C6—C7—C8	118.37 (16)	C14—C15—H15	119.9
C7—C8—C9	121.12 (17)	C16—C15—H15	119.9
C8—C9—C10	120.10 (15)	C15—C16—H16	120.3
C9—C10—C11	120.05 (15)	C17—C16—H16	120.3
C6—C11—C10	118.50 (13)	C16—C17—H17	119.8
C6—C11—C12	117.48 (12)	C18—C17—H17	119.8
C10—C11—C12	123.97 (13)	C13—C18—H18	119.7
O3—C12—C4	124.57 (13)	C17—C18—H18	119.7
O3—C12—C11	113.70 (12)	O6—C20—H201	110.1
C4—C12—C11	121.72 (13)	O6—C20—H202	110.1
C3—C13—C14	118.36 (14)	C21—C20—H201	110.1
C3—C13—C18	123.41 (13)	C21—C20—H202	110.1
C14—C13—C18	118.23 (14)	H201—C20—H202	109.5
C13—C14—C15	121.16 (16)	C20—C21—H211	109.5
C14—C15—C16	120.15 (16)	C20—C21—H212	109.5
C15—C16—C17	119.36 (17)	C20—C21—H213	109.5
C16—C17—C18	120.49 (18)	H211—C21—H212	109.5
C13—C18—C17	120.61 (15)	H211—C21—H213	109.5
O5—C19—O6	126.28 (16)	H212—C21—H213	109.5
			
C5—O2—C6—C7	−176.87 (15)	C3—C4—C5—O2	−179.57 (14)
C5—O2—C6—C11	2.1 (2)	C3—C4—C5—O4	−0.0 (2)
C6—O2—C5—O4	−178.82 (15)	C3—C4—C12—O3	−1.0 (2)
C6—O2—C5—C4	0.8 (2)	C3—C4—C12—C11	179.57 (14)
C1—O3—C12—C4	−12.7 (2)	C5—C4—C12—O3	−176.60 (15)
C1—O3—C12—C11	166.76 (13)	C5—C4—C12—C11	4.0 (2)
C12—O3—C1—O1	−79.87 (15)	C12—C4—C5—O2	−3.8 (2)
C12—O3—C1—C2	41.82 (17)	C12—C4—C5—O4	175.79 (17)
C12—O3—C1—C19	164.28 (13)	O2—C6—C7—C8	179.32 (16)
C19—O6—C20—C21	179.82 (15)	O2—C6—C11—C10	−179.62 (14)
C20—O6—C19—O5	5.1 (2)	O2—C6—C11—C12	−1.9 (2)
C20—O6—C19—C1	−175.90 (13)	C7—C6—C11—C10	−0.7 (2)
O1—C1—C2—C3	61.60 (16)	C7—C6—C11—C12	176.99 (16)
O1—C1—C19—O5	−14.1 (2)	C11—C6—C7—C8	0.4 (2)
O1—C1—C19—O6	166.88 (12)	C6—C7—C8—C9	−0.1 (2)
O3—C1—C2—C3	−58.67 (16)	C7—C8—C9—C10	0.1 (2)
O3—C1—C19—O5	100.86 (17)	C8—C9—C10—C11	−0.5 (2)
O3—C1—C19—O6	−78.16 (14)	C9—C10—C11—C6	0.8 (2)
C2—C1—C19—O5	−139.51 (16)	C9—C10—C11—C12	−176.80 (16)
C2—C1—C19—O6	41.46 (18)	C6—C11—C12—O3	179.34 (14)
C19—C1—C2—C3	−173.59 (12)	C6—C11—C12—C4	−1.2 (2)
C1—C2—C3—C4	43.95 (17)	C10—C11—C12—O3	−3.1 (2)
C1—C2—C3—C13	−84.91 (15)	C10—C11—C12—C4	176.41 (16)
C2—C3—C4—C5	160.47 (14)	C3—C13—C14—C15	−179.77 (15)
C2—C3—C4—C12	−15.2 (2)	C3—C13—C18—C17	−179.88 (15)
C2—C3—C13—C14	−64.76 (18)	C14—C13—C18—C17	0.7 (2)
C2—C3—C13—C18	115.79 (16)	C18—C13—C14—C15	−0.3 (2)
C4—C3—C13—C14	169.80 (14)	C13—C14—C15—C16	−0.4 (2)
C4—C3—C13—C18	−9.7 (2)	C14—C15—C16—C17	0.7 (2)
C13—C3—C4—C5	−71.53 (18)	C15—C16—C17—C18	−0.4 (3)
C13—C3—C4—C12	112.80 (17)	C16—C17—C18—C13	−0.4 (2)

### Hydrogen-bond geometry (Å, °)

**Table:**

*D*—H···*A*	*D*—H	H···*A*	*D*···*A*	*D*—H···*A*
O1—H101···O2^i^	0.82	2.30	2.9198 (15)	132
O1—H101···O5	0.82	2.17	2.6628 (17)	119

Symmetry codes: (i) −*x*+1/2, *y*+1/2, −*z*+1/2.
